# The Week's Work

**Published:** 1911-01-21

**Authors:** 


### The Week's Work.

### CO-OPERATION IN CONTRACTS.

A very important and instructive report has been published by the Sub-Committee appointed by the Executive Committee of King Edward's Hospital Fund to consider the advantages and possibilities of co-operation in the matter of hospital contracts. This question is one of such immense importance to all hospital managers that they may be urged to study the report itself in detail, for it contains much that is highly instructive. The members of the Sub-Committee were Mr. J. G. Griffiths (chairman), the Hon. Sydney Holland, and the Hon. Arthur Stanley. Evidence was taken from a number of witnesses representing various hospitals and from other experts in hospital finance and management. Naturally a good deal of attention was devoted to the scheme of a Central Purchasing Agency, which has been on a previous occasion described in these pages, and has been adopted by some of the New York hospitals. The Sub-Committee is very guarded in referring to this scheme, but concludes in general terms that a system of co-operative purchase, if a practicable one can be evolved, may prove of much advantage to groups of small hospitals. For medium-sized institutions the benefits are much more problematical, while for the largest there would be none at all, save in respect of a few special items. The report proceeds as follows: "Our general conclusion is that it would not be profitable to recommend the establishment of any combined scheme for the whole of the hospitals of London, or for the larger hospitals taken by themselves. We would suggest rather the consideration of a scheme of co-operation by groups of hospitals, each composed of several small or moderate-sized institutions acting in combination with one or more larger hospitals. For obvious reasons, one of the determining factors in the formation of such groups would be geographical situation. We think that arrangements of this sort would be likely to prove to the mutual advantage of most, if not all, of the institutions concerned." The practical issue, then, is that the Central Purchasing Agency is not regarded as suitable for the needs of London; yet it is to be noted that many of the strong points of that American innovation are incorporated with the recommendations now published.

### GEOGRAPHICAL LIMITATIONS.

The Sub-Committee has, in our opinion, discovered and proved the weak spot in the Central Depot scheme to be that of distribution, the difficulty and cost of which are caused chiefly by the question of geography. If London were a city like New York, built on a cramped and limited site incapable of expansion, with every hospital within a comparatively short distance of the centre, this difficulty would be much less; but as things are, it forms one of the rocks upon which the scheme must split. The cost of establishing a Central Purchasing Agency, of staffing it with experts—unless it attracted first-rate men by first-rate salaries it would be a hopeless failure—and of providing means of transport for supplying the requisitions arising from the various hospitals so widely scattered over London's enormous area; the cost of all this would in all probability come to more than the profits which could be made by buying in greater bulk. It is most satisfactory to note that the Fund is recommended not to put any pressure on hospitals to induce them to enter upon mutual co-operation, while at the same time it is very pertinently observed that evidences of economy and careful management thus achieved may well be taken into account in preparing the annual distribution of the awards granted by the Fund. That which hospital managers, particularly those of small hospitals, may usefully bear in mind is that the question of thrift and efficiency in the financial affairs of their respective institutions is left to their own initiative; and that if, by taking counsel with neighbouring hospitals, they can effect economies by contracting for joint supplies, the King's Fund will view the results with a benevolent and appreciative eye. Further, the forms of tender at present in vogue are found to vary very widely; some are excellent, others very vague and ill-drawn. The Sub-Committee urges the adoption of model forms, not necessarily as standards to be slavishly copied and rigidly adhered to, but as specimens to illustrate the most important points to be borne in mind when calling for tenders. In submitting to the Fund such schedules of tenders, based upon the experience of those hospitals where the points in view have been most carefully studied, the Sub-Committee has earned the gratitude of every hospital secretary and hospital board in the kingdom; and it is to be hoped that its labours may be put to practical and fruitful use whenever contracts have to be entered into or renewed.

### THE CONSTRUCTION OF HOSPITALS IN THE TROPICS.

In a recent issue of *Het Ziekenhuis* Dr. R. Römer gives some interesting details on the above-mentioned subject, taking as his model the excellent new hospital of the Deli Company at Medan-Deli. Dr. Römer lays deserved stress upon the differences between hospital construction in temperate and in tropical countries, but it is evident that the main points which should guide those responsible for the planning of a hospital are the same everywhere. A matter of importance, for example, is the disposal of refuse and night soil. In civilised countries the architect has no great difficulty: he can either turn his drains into the main drainage system, or construct septic tanks with accessory refuse destructors. In the tropics, however, the septic tank method is an abomination, and reliance has to be placed on the destructor. Dr. Römer hints that a hospital site should be close to the banks of a good running stream, into which the sewerage of the institution may be drained. This appears to us to be a bad principle. The only way to get rid of hospital refuse is to render it perfectly harmless to health by incinerating or otherwise destroying it by heat. Nowadays excellent destructors, portable, light, and thoroughly reliable, can be obtained from any reputable firm, and a hospital in the colonies should not hesitate to invest in one of these aids to cleanliness. The initial expense will be fully justified by the additional security obtained.

### THE HOSPITAL PLAN IN WARM CLIMATES.

The ideal hospital for the tropics is one of the single-story pavilion type, with double-roof ventilation. Buildings on the corridor system, or double-story pavilions, are to be condemned owing to the difficulty of obtaining efficient ventilation. The internal arrangements of the wards offer scope for the exercise of much ingenuity on the part of the superintendent and the staff. First, there is the question of water-supply. In the tropics the usual hospital average is given at 75 litres per patient, which appears rather high when one considers that the average for drinking purposes is usually estimated at 3 litres per head for Europeans and 2 litres per head for natives. Dr. Römer recommends the use of a hospital pump to work independent deep-level wells, and warns against the use of river water, both for bathing and drinking purposes, on account of the danger of ankylostomiasis and other parasitic diseases. The Deli-Medan Hospital, of which plans and very beautiful illustrations from photographs are given, has a capacity of 450 patients, and is ideally simple in construction. The wards are cement floored, and some of them are mosquito-proof—the cholera ward especially so—owing to the care which has been taken to close every opening with fine netting. The hospital has a special pathological and bacteriological department, and to it is attached a small leprosy asylum for about one hundred patients. It is interesting, as a side issue, to hear that the patients object to pillows; nor are mattresses much in favour. Very acutely ill patients are given an ordinary mattress fitted with coir, but as soon as they improve they attempt to get rid of this, preferring to rest on a hard, smooth surface; bed-sores are rarely seen. A specially constructed steel-spring mattress is figured. The article will repay study by those who are interested in hospital construction.

### HOW TO HELP THE HOSPITALS.

The financial problem, How to raise funds for voluntary hospitals? was discussed by Miss Jaquith, superintendent of the Worcester Memorial Hospital, Mass., at the recent Congress of the American Hospitals' Association. Her paper is printed in the last number of the *International Hospital Record*, and deserves to be read by all hospital committee men. Miss Jaquith concludes that there are numerous ways of raising small sums for current expenses, but very few really useful methods of raising a large amount to cover a deficit. In an appeal it is of the utmost importance that the institution should aim at securing a really influential committee. The objections to a "one-man appeal" are obvious. It throws a vast strain on the energy of the individual who has been selected to canvass for funds, while it robs the institution of the help which other influential supporters might have brought had they been conjoined with the appellant in soliciting aid from the public. A large and influential committee can work in many directions: one man or woman, however eminent, only in one. The ideal method would, of course, be to select a well-known personality as Chairman of the Appeal Committee, and to give him, or her, the help of a number of active and influential supporters, each of whom would be a nucleus for a band of friends and workers. In this manner the greatest good would be accomplished with the minimum expense of energy. Miss Jaquith points out that we do not put our business claims so prominently forward as we might do. A good hospital deserves the support of every employer of labour just as much as it deserves the support of every working man for obvious reasons. Business men, as a rule, are always willing to acknowledge this, and to give financial assistance during the hard days. But, from the hospital's point of view, it is eminently more satisfactory to have a prominent head of a large firm as an active member of an Appeal Committee than merely to get that firm to contribute a substantial cheque to the funds of the institution.

### THIS WEEK'S DRUG MARKET.

A fair business has been done, and on the whole prices are well maintained, with few actual changes in quotations. The announcement of the advance in the price of santonin has been withdrawn, so that quotations are the same as they were before the announcement; it is anticipated, however, that before long prices will be definitely advanced. Ergot of rye remains very scarce, and prices are still tending upwards. Siam gum benzoin fetched very high prices at the recent drug sales. Grey Jamaica sarsaparilla is dearer. Balsam tolu is tending higher in price, and asafœtida rather lower.

